# Generation of a whole-brain hemodynamic response function and sex-specific differences in cerebral processing of mechano-sensation in mice detected by BOLD fMRI

**DOI:** 10.3389/fnins.2023.1187328

**Published:** 2023-08-28

**Authors:** Hui-Fen Chen, Henriette Lambers, Nina Nagelmann, Martin Sandbrink, Daniel Segelcke, Esther Pogatzki-Zahn, Cornelius Faber, Bruno Pradier

**Affiliations:** ^1^Clinic of Radiology, Translational Research Imaging Center (TRIC), University of Münster, Münster, Germany; ^2^Department of Anesthesiology, Intensive Care and Pain Medicine, University of Münster, Münster, Germany

**Keywords:** hemodynamic response function (HRF), Blood-oxygenation-level-dependent (BOLD), mechanical stimulation, sex differences, mouse fMRI

## Abstract

BOLD fMRI has become a prevalent method to study cerebral sensory processing in rodent disease models, including pain and mechanical hypersensitivity. fMRI data analysis is frequently combined with a general-linear-model (GLM) -based analysis, which uses the convolution of a hemodynamic response function (HRF) with the stimulus paradigm. However, several studies indicated that the HRF differs across species, sexes, brain structures, and experimental factors, including stimulation modalities or anesthesia, and hence might strongly affect the outcome of BOLD analyzes. While considerable work has been done in humans and rats to understand the HRF, much less is known in mice. As a prerequisite to investigate mechano-sensory processing and BOLD fMRI data in male and female mice, we (1) designed a rotating stimulator that allows application of two different mechanical modalities, including innocuous von Frey and noxious pinprick stimuli and (2) determined and statistically compared HRFs across 30 brain structures and experimental conditions, including sex and, stimulus modalities. We found that mechanical stimulation lead to brain-wide BOLD signal changes thereby allowing extraction of HRFs from multiple brain structures. However, we did not find differences in HRFs across all brain structures and experimental conditions. Hence, we computed a whole-brain mouse HRF, which is based on 88 functional scans from 30 mice. A comparison of this mouse-specific HRF with our previously reported rat-derived HRF showed significantly slower kinetics in mice. Finally, we detected pronounced differences in cerebral BOLD activation between male and female mice with mechanical stimulation, thereby exposing divergent processing of noxious and innocuous stimuli in both sexes.

## Introduction

Blood oxygenation level dependent (BOLD) functional magnetic resonance imaging (fMRI) has become a prevalent method to study evoked neuronal circuits in rodent disease models (Jonckers et al., 2015; Xu et al., 2022). It is frequently combined with a general-linear-model (GLM) -based analysis to detect BOLD responses, which uses the convolution of the hemodynamic response function (HRF) with the stimulus paradigm for statistical analysis of fMRI data (Friston et al., 1998; Henson and Friston, 2007). However, several studies indicate that the HRF differs across experimental factors, including anesthesia, stimulation modalities, brain structures, sexes, and species, and hence might strongly affect the outcome of BOLD analyses (Duque et al., 2017; Lambers et al., 2020; Wang et al., 2020). Therefore, the determination of species or even data-specific HRF has been acknowledged to be important and is increasingly applied in animal fMRI studies (Schroeter et al., 2014; Jung et al., 2019, 2021; Dinh et al., 2020; You et al., 2021). While much work has been done to describe the temporal dynamics of HRF in rats (Lambers et al., 2020), such systematic comparisons are rare in mice, and yet, incomplete regarding different experimental factors (Schlegel et al., 2015).

Since investigation of sensory processing is vital to many brain disorders, different fMRI task (stimulus) modalities have been developed and applied to mouse fMRI studies; these include electrical or heat stimulation of the glabrous paw skin (Nair and Duong, 2004; Adamczak et al., 2010; Neely et al., 2010; Schroeter et al., 2014; Bosshard et al., 2015; Schlegel et al., 2015, 2018; Reimann et al., 2016; Shim et al., 2018; Jung et al., 2019, 2021; Just et al., 2020; Moon et al., 2021; You et al., 2021), or the whisker pad (Desjardins et al., 2019; You et al., 2021), visual, olfactory or auditory (Niranjan et al., 2016, 2017; Blazquez Freches et al., 2018; Boido et al., 2019; Chen et al., 2020; Dinh et al., 2020; Komaki et al., 2020; Zhao et al., 2020; Pradier et al., 2021; Lungu et al., 2022), and optogenetic stimulation (Kahn et al., 2011; Salvan et al., 2021; Wank et al., 2021). However, differences in modality-specific HRF responses are difficult to compare since different stimulation modalities will intrinsically activate a distinct subset of brain structures that are specific to modality processing. Furthermore, many of the above-mentioned studies predominantly focused on cortical BOLD responses and determined cortical HRFs and hence it is unknown whether differences in BOLD responses exist in different brain structures in mice. Yet, several studies using techniques other than BOLD fMRI (e.g., intrinsic optical imaging, arterial spin labeling (ASL), immunohistochemistry) suggest differences in blood flow dynamics and blood vessel density in different brain structures, including cortex, hippocampus and thalamus (Hayward et al., 2011; Wiesmann et al., 2016, 2017; Bernier et al., 2021). Moreover, other factors, including stimulation paradigm (Schlegel et al., 2015; Lambers et al., 2020) and anesthesia (Schroeter et al., 2014; Schlegel et al., 2015) are known to modulate the HRF significantly and, together with brain structures, are likely to be more critical factors for the HRF than the stimulus modality *per se.*

Despite overall sex differences in cerebral processing in small animals and humans (Levin et al., 1998; Baran et al., 2010; Dumais et al., 2017; Bloch et al., 2021), only a few studies have systematically investigated sex differences in the temporal progression of the BOLD response or parameters of the hemodynamic response in humans (Marumo et al., 2009; Squair et al., 2020) or rats (Lambers et al., 2020; Wang et al., 2020). While sex differences are probably involved in the BOLD response through the interaction of, for example, estrogen with the sympathetic nervous system resulting in vasoconstriction (Duque et al., 2017), studies with large human cohorts (*n* > 100) did not detect differences in parameters of the hemodynamic response (Squair et al., 2020; Leacy et al., 2022). Yet, to the best of our knowledge, no such studies have been performed in mice related to this.

Additionally, sensitization of skin-resident mechanoreceptors and mechanical hypersensitivity as a central phenomenon is specific to different human pain disorders and involves different mechanisms (Arcourt et al., 2017). To investigate these mechanisms, withdrawal of the paw in response to a noxious thermal or mechanical stimulus is typically measured in rodents to assess pain-related behavior (González-Cano et al., 2020). The withdrawal response to von Frey filaments (blunt, rather innocuous) is the current gold standard for measuring mechanical hypersensitivity in rodent pain models and hence, is frequently used (Deuis et al., 2017; Segelcke et al., 2019) and enables detection of relevant sex differences in rodents (Segelcke et al., 2021, 2023). More rarely, supra-threshold mechanical stimuli using pinpricks (sharp, noxious) are used to investigate mechanisms of clinically significant hyperalgesia, e.g., in postoperative pain (Segelcke et al., 2019).

Here, we demonstrate cerebral processing via BOLD analysis of mechanical hind paw stimulation in mice; we applied innocious and noxious mechanical modalities to the plantar aspect of the hind paw in male and female mice under combined isoflurane/medetomidine anesthesia (Pradier et al., 2021) and analyzed differences in temporal progression of HRF in 30 structures covering the whole brain. Finally, we investigated BOLD responses to map sex differences in sensory processing during innocuous and noxious mechanical stimulation.

## Materials and methods

### Animals

**Figure 1:**
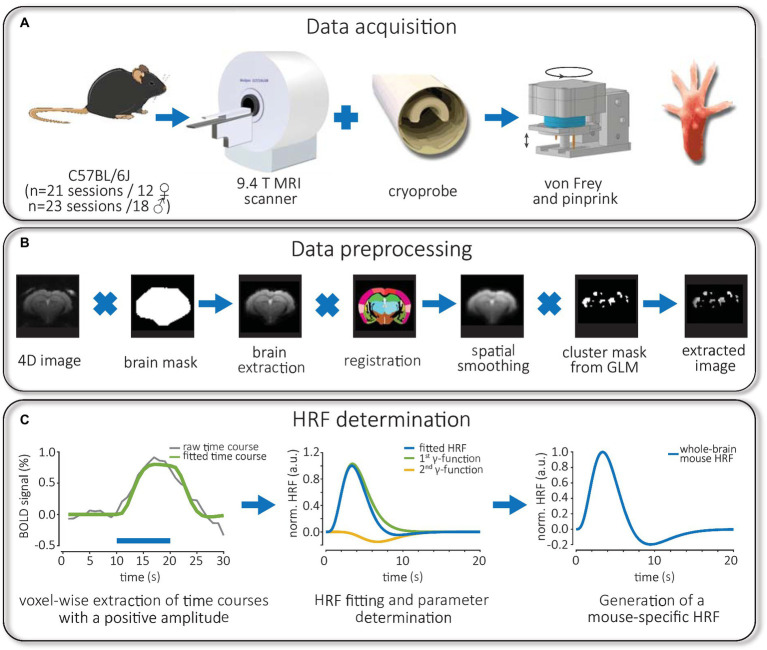
Workflow of experiments: fMRI data acquisition, image preprocessing and HRF measurement. **(A)** C57Bl/6 J mice of both sexes were used for fMRI experiments in a 9.4T small animal scanner equipped with a cryoprobe. Using a rotating stimulator, mice were mechanically stimulated at the same position of the right hind paw using calibrated von Frey filaments or pinprick. **(B)** Preprocessed 4D images were skull stripped via brain masks, registered to a mouse atlas and smoothed spatially. Later, individual time courses from significantly activated voxels were extracted using binary cluster masks obtained from FIR-based GLM analysis. **(C)** The time course of positive BOLD responses was averaged for each brain structure and fitted. Then, the convolution of the HRF, consisting of two gamma functions, with the stimulation paradigm, was fitted to the BOLD response. Finally, the parameters of a whole-brain mouse-specific HRF were obtained for an implementation in SPM.

All animal experiments were carried out according to the German Animal Welfare Act, and were approved by the State Agency for Nature, Environment and Consumer Protection North Rhine-Westphalia, Germany (LANUV, approval ID 81–02.04.2018.A013). C57BL/6J mice (18 males and 12 females weighing 20–30 g, 8–10-week-old) were used in this study (Figure 1A, left). Animals were obtained from the local animal facility, housed in groups of 2–5 in individually ventilated cages (IVC), kept at a 12/12 h light/dark cycle, and had access to standard diet and water *ad libitum*. Both sexes were housed in the same rack in the same room. Estrus cycles of females were not controlled because female mice, when housed in groups or with males in the same room, have a synchronized cycle (van der Lee and Boot, 1955; Whitten, 1956, 1957; Whitten et al., 1968). Furthermore, a meta-analysis demonstrated similar variability in data (behavioral, morphological, physiological, and molecular traits) from male and female mice (Prendergast et al., 2014). Furthermore, different phases of the estrous cycle did not affect sensory perception/nociception in C57BL/6J female mice (Meziane et al., 2007).

### MRI measurements

All fMRI measurements followed a procedure of anesthesia similar as described previously (Pradier et al., 2021). Briefly, the fMRI measurements were performed using a 9.4T Bruker Biospec 94/20 small animal MRI scanner (Bruker Biospin GmbH, Ettlingen, Germany) with a cryogenic quadrature RF surface probe (Bruker) for signal transmission and reception (Figure 1A, middle). Prior to fMRI measurements, mice were first anesthetized with 5% isoflurane (ISO, Baxter) in 75% air and 25% oxygen and then supplied with 1.5% ISO during preparation of the animals on the cradle. After global and local shimming of the brain using MapShim (Bruker), task fMRI measurements were performed using a single-shot gradient EPI sequence (TR/TE 1,000/18 ms, matrix 76 × 66, resolution 200 × 200 μm^2^, 18 slices, slice thickness 0.5 mm, flip angle 60°, 620 repetitions) under subcutaneous (s.c.) medetomidine (MED, Dormitor^®^, Orion Pharma) sedation combined with 0.2% ISO, which was reduced from 1.0 to 0.2% during the first 20 min following MED bolus injection (0.1 mg/kg bolus, 0.2 mg/kg per hour of continuous infusion). This anesthetic regimen produces a stable brain state, functional connectivity and reliable BOLD activation in mice (Pradier et al., 2021). The first task fMRI experiment started 50 min after initial MED bolus injection. After fMRI experiments, mice were injected with atipamezole (Antisedan^®,^ Orion Pharma) to counteract MED at the same concentration and volume as used for the previous MED infusion.

One week before starting the first functional imaging experiment, mice were habituated to the combined anesthesia/sedation regimen for 1 h on the bench to avoid confounding effects during longitudinal fMRI measurements. During the imaging session, we recorded the respiration rate of the animals using an MR-compatible monitoring system (Small Animal Instruments, Inc., New York, United States) and observed 124 ± 3 in male and 142 ± 3 breaths per minute for female mice (mean ± S.E.M). Using two-way ANOVA, we detected a sex difference in respiration rate [*F*_(1, 80)_ = 17.31, *p* < 0.0001]. A rectal fiber optic probe was used to monitor the body temperature (Neoptix Inc., Quebec, Canada), which was maintained at 36.2 ± 0.1°C (mean ± S.E.M) using a tube system positioned underneath the animal that was connected to a water bath.

### Mechanical stimulation

We developed a new MR-compatible mechanical stimulation device that—through rotational movement between fMRI scans—allows stimulation with multiple mechanical modalities during one imaging session in mice (Figure 1A, right). Modalities applied were von Frey filaments (vF) and pinprick (pp). Stimuli were calibrated to a force of 200 mN and targeted to the plantar aspect of the right hind paw. Therefore, our setup resulted in four groups: female vF, female pp, male vF and male pp. The order of modalities (vF—pp, pp—vF) was balanced over different mice. Task fMRI scans were performed using a 20-block paradigm with 10 s stimulation/20 s rest, a stimulation frequency of 1 Hz and a pulse duration of 0.5 s. Between task fMRI scans, mice were allowed a 20-min period without stimulation.

### fMRI data processing and extraction of BOLD time course

**Figure 2:**
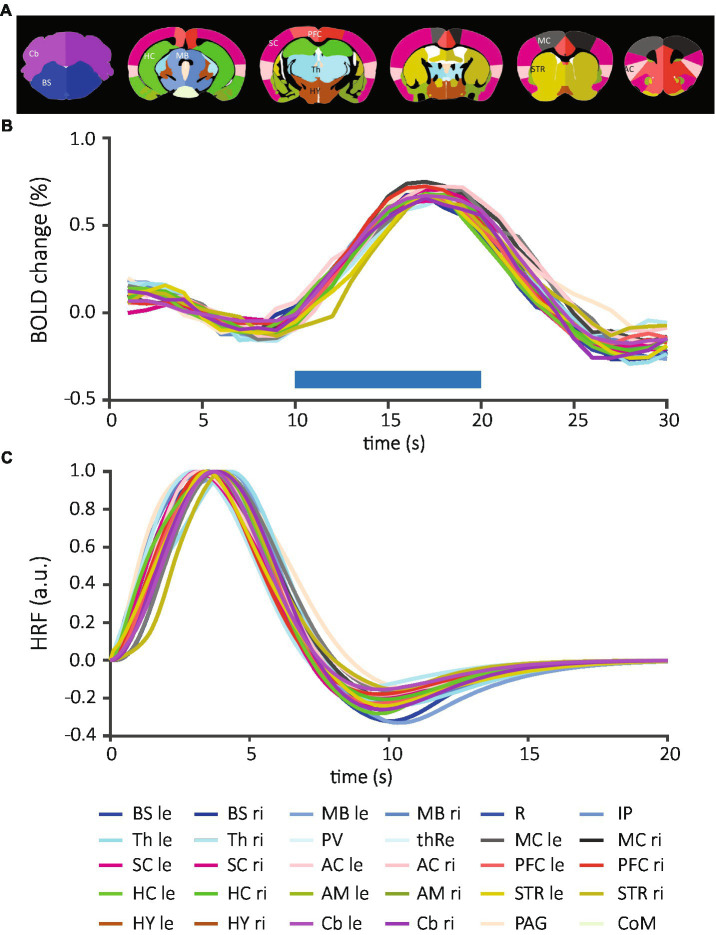
Brain structure-specific BOLD responses and normalized mean HRFs averaged across all data sets. **(A)** The anatomical atlas included 30 brain structures and was registered to each EPI image. **(B)** After extraction of time courses and determination of positively activated voxels, individual time courses of each structure were measured per animal and then averaged across all data sets. Blue horizontal bar demonstrates the onset and duration of the stimulation. **(C)** The time course of BOLD responses was fitted to the convolution of the “canonical” HRF and the stimulation paradigm. From these fits, HRFs were calculated for each brain structure. Each line represents an individual brain structure according to the mouse atlas. Bilateral structures: BS, brainstem; MB, midbrain; Th, thalamus; MC, motor cortex; SC, sensory cortex; AC, association cortex; PFC, prefrontal cortex; HC, hippocampus; AM, amygdala; STR, striatum; HY, hypothalamus; Cb, cerebellum; le, left; ri, right; medial structures, R, raphe nucleus; IP, interpeduncular nucleus; PV; paraventricular thalamic nucleus; thRe, nucleus reuniens of the thalamus; PAG, periaqueductal gray; CoM, corpora mammillaria.

Data processing (Figure 1B) of task fMRI experiments started with conversion from DICOM to NIfTI format using MRIcroGL.^1^ We discarded the first 20 scans of each measurement to avoid pre-steady-state artifacts. The software SPM12 was used for MR data preprocessing (Penny et al., 2007), increasing voxel size by a factor of 10, slice timing correction, realignment and spatial smoothing with a 4*4*10 mm Gaussian kernel. GLM analysis was performed using the 9^th^ order of the finite impulse response (FIR) basis, which consisted of a set of nine boxcar functions (Penny et al., 2007). Significantly activated voxels found by GLM analysis (p_uncorr_ < 0.05, cluster size > 5 voxels) were exported as a binary mask for subsequent preprocessing to MagnAn 2.5 (BioCom, Uttenreuth, Germany) as described previously (Pradier et al., 2021). For co-registration, a house-build anatomical atlas consisting of 30 brain structures (Figure 2A) that was based on the Mouse brain atlas (Franklin and Paxinos, 2008; Atlas, 2011) was used (Supplementary Table S1). For this purpose, the preprocessed fMRI data were first brain-extracted and subsequently registered affine, with 6 degrees of freedom, using the regibox module in MagnAn for co-registration and spatial normalization. fMRI data were multiplied with the binary mask (obtained from BOLD map of the GLM analysis) and the label mask to allow brain structure-specific time series extraction from activated voxels as described previously (Pradier et al., 2021). Then, BOLD activation probability per group was calculated from the co-registered and spatially normalized BOLD maps.

### Generation and calculation of whole-brain mouse HRFs

The calculation of mouse-specific HRFs was conducted similar to a procedure described earlier for rats (Lambers et al., 2020). First, the time courses were temporally smoothed by a moving average with 5 s and then we determined whether the BOLD signals had a positive or negative time course on a voxel-by-voxel basis; to this end, we averaged the time series over 20 repetitions and normalized it to the baseline (Figure 1C, left). To allow successful fitting of the HRF using two gamma functions, time courses needed to meet 2 criteria: (1) the amplitude of the BOLD response had to be at least 0.6% and (2) the BOLD signal had to decay by at least 40% following the maximum peak. Since the FIR model is not biased toward the sign of the BOLD response, we found that 8.36 ± 1.15% of activated voxels had positive time courses according to the above criteria. Next, time courses were averaged for each brain structure; note that only brain structures were included if they contained more than four voxels with a positive time course. Then the convolution of stimulation paradigm and HRF was fitted to the averaged time courses for each brain structure and measurement (Figure 1C, middle) by adjusting the parameters (A,b,p1,p2,V) of the HRF, consisting of two gamma functions, as described previously (Lambers et al., 2020):


$$HRF(\tau )=\;A\cdot e^{-bt}\cdot \left(\frac{b^{p_{1}}}{\Gamma \left(p_{1}\right)}\cdot t^{p_{1}-1}-\frac{b^{p_{2}}}{V\cdot \Gamma \left(p_{2}\right)}\cdot t^{p_{2}-1}\right).$$

Fitting results were excluded (21.05%) when they showed a normalized mean squared error (MSE) > 0.1 compared to the original BOLD time course, or when the fitted BOLD time course had an early onset (<10 s) that started prior to the stimulation.

To examine whether there were significant differences between HRFs of two modalities or brain structures, we performed functional *t*-tests (Ramsay et al., 2009) as previously described (Lambers et al., 2020)^2^ using 10,000 permutations (Eklund et al., 2011). In detail, the test examined whether two samples *x* and *y*, both composed of several HRFs, differ. To compare the samples, first the *t*-value t(τ) was calculated for each time point τ of the functions using the sample sizes $n_{x}$ and $n_{y}$ , the mean values and *HRFsx* and ${\bar{HRFs}}_{y}\left(\tau \right)$ as well as the variances $\mathrm{var}\left[HRFs_{1}\left(\tau \right)\right]$ and $\mathrm{var}\left[HRFs_{2}\left(\tau \right)\right]$ :


$$t\left(\tau \right)=\frac{\left|{\bar{HRFs}}_{x}\left(\tau \right)-{\bar{HRFs}}_{y}\left(\tau \right)\right|}{\sqrt{\frac{1}{n_{x}}\mathrm{var}\left[HRFs_{1}\left(\tau \right)\right]+\frac{1}{n_{y}}\mathrm{var}\left[HRFs_{2}\left(\tau \right)\right]}}$$

Subsequently, the maximum of the *t*-values contained in t(τ) was determined. To assess whether the maximum *t*-value represents a significant difference, a permutation test was performed. HRFs were subjected to 10,000 permutations and the maximum *t*-value was determined for each permutation. Then the number *N* was calculated, which shows how often the maximum *t*-value of any permutation exceeded the maximum *t*-value of the original distribution. Using the ratio of the number *N* and the number of performed permutations, the probability was calculated that the maximum *t*-value of the original distribution is smaller than the maximum *t*-value of any permutation. This probability represented the *p*-value of the functional *t*-test.

To investigate differences in HRFs of the 30 brain structures, we combined data sets from 4 groups and compared all structures using the functional *t*-test (Supplementary Table S2). We also performed this analysis within each of the four groups (Supplementary Tables S3, S4). Here, thresholds for statistical significance (*p* < 0.05) were adjusted for the number of comparisons using the Bonferroni correction, resulting in adjusted *p*-values. Only comparisons with sample sizes *n* ≥ 6 were considered for statistical testing. Since statistical testing did not show significant differences between brain structures, we pooled and averaged HRFs at the whole brain level and next, compared the effect of modality and sex. Since this comparison did not show significant differences we calculated the mean of the mouse HRFs normalized to the maximum and obtained parameters of the whole brain mouse HRF for an implementation in SPM12 (Figure 1C, right). Finally, to investigate whether HRFs of mice differs from those of rats, we compared HRFs from the left somatosensory cortex (SC) in male mice to those obtained from the primary sensory hind limb cortex in male rats measured in a previous study (Lambers et al., 2020) during mechanical stimulation. Comparison between mouse and rat HRFs were performed using the functional *t*-test as detailed above.

### Assessment of the performance of GLM using the mouse HRF

To assess the detection performance of the whole-brain mouse HRF, the determined parameters of the mouse HRF were inserted in the “canonical” basis set as implemented in SPM. The canonical basis set is composed of a double gamma HRF and their time and dispersion derivatives, which are convoluted with the stimulation paradigm. When only the convolution of the paradigm and the HRF is used for analysis, the basis set is called 1^st^ order “canonical” basis set. When both derivatives are used, the set is referred to as 3^rd^ order “canonical” basis set. The FIR basis set models the BOLD response with a series of consecutive box functions. The number of boxes corresponds to the order the set. We compared GLM analyses with varying basis sets: mouse and rat HRF with 1^st^ and 3^rd^ order, and FIR with 9th order; the MATLAB code allowing implementation of the whole brain mouse and cortical rat HRF in SPM is available at https://github.com/TheFaberLab/mouse_HRF and https://github.com/TheFaberLab/rat_HRF. We determined significantly activated voxels (p_uncorr_ < 0.05, cluster size >5 voxels) and exported these as binary masks. Masks were then averaged per group to receive the activation probability. Next, we saved the probability map via MRIcroGL and then calculated the dice index as metric for similarity of the patterns of activation probability for each model using the mouse HRF with the 1^st^ order as ground truth. Finally, we calculated the numbers of voxels with a positive time course to further characterize the GLM performances.

### Statistical analysis

To test for global significant differences in between activated brain structures, sexes and modalities, the number of activated voxels per structure and animal was calculated for each group and compared using three-way ANOVA. Next, we performed two-way ANOVA followed by Tukey’s *post-hoc* test to identify which brain structure significantly contributed to the differences observed in sex or modalities. For the correlation analysis of RR and BOLD activation in male and female mice, we performed a linear regression analysis in the contralateral SC.

### Software

For the operation of the 9.4T MRI scanner, ParaVision 5.1 was used. For pre- and post-processing of mouse fMRI data and extraction of BOLD time courses on a voxel-by-voxel basis, MRIcroGL, SPM12 in the MATLAB environment (The MathWorks, Inc., Natick, Massachusetts, United States) and MagnAn (BioCom, Uttenreuth, Germany) were used as described in the previous sections. All ANOVA tests and graphs were performed with Prism 8.0.2 (GraphPad Software, San Diego, CA, United States).

## Results

### Mechanical stimulation leads to brain-wide BOLD signal changes

To assess potential difference in HRF between sex, stimulation modality and brain structure, we extracted the BOLD responses for each brain structure from 88 fMRI scans on a voxel-by-voxel basis. We detected positive BOLD responses in 24 of 30 brain structures (Supplementary Figures S1A, S2); these included periaqueductal gray (PAG), and both hemispheres of brainstem (BS), midbrain (MB), thalamus (Th), motor cortex (MC), somatosensory cortex (SC), association cortex (AC), prefrontal cortex (PFC), striatum (STR), and cerebellum (Cb). SC, PFC, HC, and Cb were among the structures with the highest number of detected signals. Only few positive BOLD responses were extracted from paraventricular nucleus of the thalamus (PV) and the hypothalamus (HY), while no positive responses were detected in amygdala (AM), corpora mamillaria (CoM), raphe nucleus (R), and interpeduncular nucleus (IP). Overall, we found similar structure-specific BOLD time courses in female (Supplementary Figure S1A, top) and male mice (Supplementary Figure S1A, bottom) after vF and pp. stimulation. On average, the time to peak after stimulation onset was 6.9 ± 0.1 s (mean ± S.E.M) and the amplitude of the BOLD was 0.838 ± 0.003% (mean ± S.E.M, Figure 2B).

### HRFs are not different across brain structures, sex or stimulation modality

We fitted the convolution of the “canonical” HRF and the stimulation paradigm to the BOLD time courses (Figure 1C, left). From these fits, HRFs were calculated for each brain structure across all data sets (Figure 2C and Supplementary Figure S3) and for individual experimental groups (Supplementary Figure S1B). Following quality control (see material and methods), 484 HRFs were included for statistical comparisons between brain structures, sexes and stimulations. After Bonferroni correction, we did not detect significant differences between brain structures when all data sets were combined (210 comparisons, adjusted threshold for significance: 0.0002; Supplementary Table S2), or separated by each of the four groups (male pp, 10 comparisons, threshold_adj_: 0.005; male vF, 6 comparisons, threshold_adj_: 0.0083; female pp, 78 comparisons, threshold_adj_: 0.0006; female vF, 78 comparisons, threshold_adj_: = 0.0006; Supplementary Tables S3, S4). Importantly, this shows that for HRFs with a positive sign, neither side of stimulation (contra- vs. ipsilateral) nor brain structure (e.g., cortical vs. subcortical) impacted HRF kinetics.

**Figure 3:**
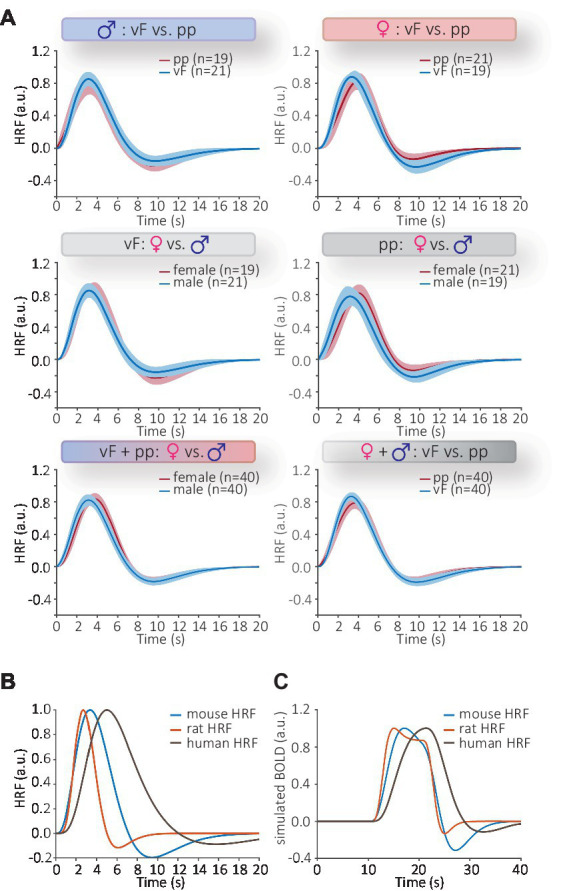
Statistical comparison of HRFs using functional *t*-test. **(A)** *Top*: comparison of modalities in male (right) and female (left) mice; *middle*: comparisons of sex following vF (*left*) and pp. (*right*) stimulation *bottom left*: comparison of all male and female HRFs; *bottom right*: comparisons of all HRFs following vF and pp. stimulation. No significant differences were found for these six comparisons. Shaded areas of the HRFs show the confidence intervals. The number of datasets per group is represented by n. **(B)** The whole-brain mouse HRF (blue) measured from 88 data sets versus the cortical rat HRF (red) and the default human HRF (brown, Penny et al., 2007). The mouse and rat HRFs both have a faster progression than the human canonical HRF. **(C)** The differences in temporal kinetics narrowed when HRFs were used to model BOLD signals. This was achieved by convolving the HRF and the stimulation paradigm. BOLD responses modeled with mouse or rat HRFs were similar, whereas BOLD signals modeled using the human HRF had slower kinetics.

Next, we investigated whether sex or modality would affect HRFs and performed between-group comparisons for each brain structure. Again, no significant differences in modality-driven HRFs were found (data not shown). Finally, we averaged time courses across all structures to generate one HRF for each data set (Figure 3A) and compared between groups: no statistically significant difference was detected (Table 1). Together, these data show that the mouse HRFs did not significantly differ between hemispheres or brain structures and were not affected by sex or stimulation.

**Table 1:** Functional *t*-test between experimental groups.

Data 1	*n*	Data 2	*n*	*p*
Male vF	21	Male pp	19	0.6338
Female vF	19	Female pp	21	0.0510
Male vF	21	Female vF	19	0.5990
Male pp	19	Female pp	21	0.1988
Male	40	Female	40	0.1424
pp	40	vF	40	0.1153

We averaged time courses across all brain structures to generate one HRF for each individual animal and compared between groups to investigate whether sex or modality differences existed.

### Species differences in temporal progression of HRF

Since no differences had been detected between mouse HRFs, we averaged all HRFs obtained from 88 datasets over all brain structures and groups, to generate a whole-brain mouse-specific HRF (Figure 3B, blue line). A comparison of the averaged HRF from mouse (this study), rat (Lambers et al., 2020) and human (SPM) showed distinctly different kinetics (Figure 3B and Supplementary Figure S4). The time to peak of mouse HRF occurred at 3.35 ± 0.10 s with the time to undershoot at 9.12 ± 0.10 s (mean ± S.E.M). The full width at half maximum (FWHM) of the mouse HRF was 3.25 ± 0.04 s (mean ± S.E.M) and lies between that of rats (2.3 s) and humans (5.3 s) HRF. When we modeled BOLD responses from these HRFs (i.e., convolving HRF and stimulation paradigm, Figure 3C), the differences in temporal kinetics became smaller; BOLD responses modeled with mouse or rat HRF showed a high temporal concordance, whereas the response modeled with human HRF differed substantially from the rodent models. Next, we statistically investigated species differences between mice and rats, and compared our data with those reported earlier obtained from male Sprague–Dawley rats (Lambers et al., 2020). For reasons of comparability, we studied differences between HRFs obtained only from the somatosensory cortex following mechanical stimulation in male mice and statistically evaluated differences between vF and pp in mice vs. low and high mechanical stimulation intensity in rats. We found pronounced species differences in HRFs following mechanical stimulation (Table 2). The brain-wide mouse HRF was characterized by the dispersion parameter b = 0.9, the peak parameters p1 = 4.5, p2 = 7.9 and the ratio parameter V = 1.8. Next, these parameters were implemented in SPM to perform GLM analysis.

**Table 2:** Functional *t*-test between species.

Data set 1 (this study)	*n*	Data set 2	*n*	*p*
Male mouse mechanical stimulation	26	SD rat male mechanical stimulation	16	0.0007

Species differences in HRFs of S1HL during mechanical stimulation, revealed by comparing our data from mice (data set 1) with those obtained earlier from male rats (data set 2, Lambers et al., 2020).

### Assessment of BOLD signal detection of mouse HRF-based GLM

**Figure 4:**
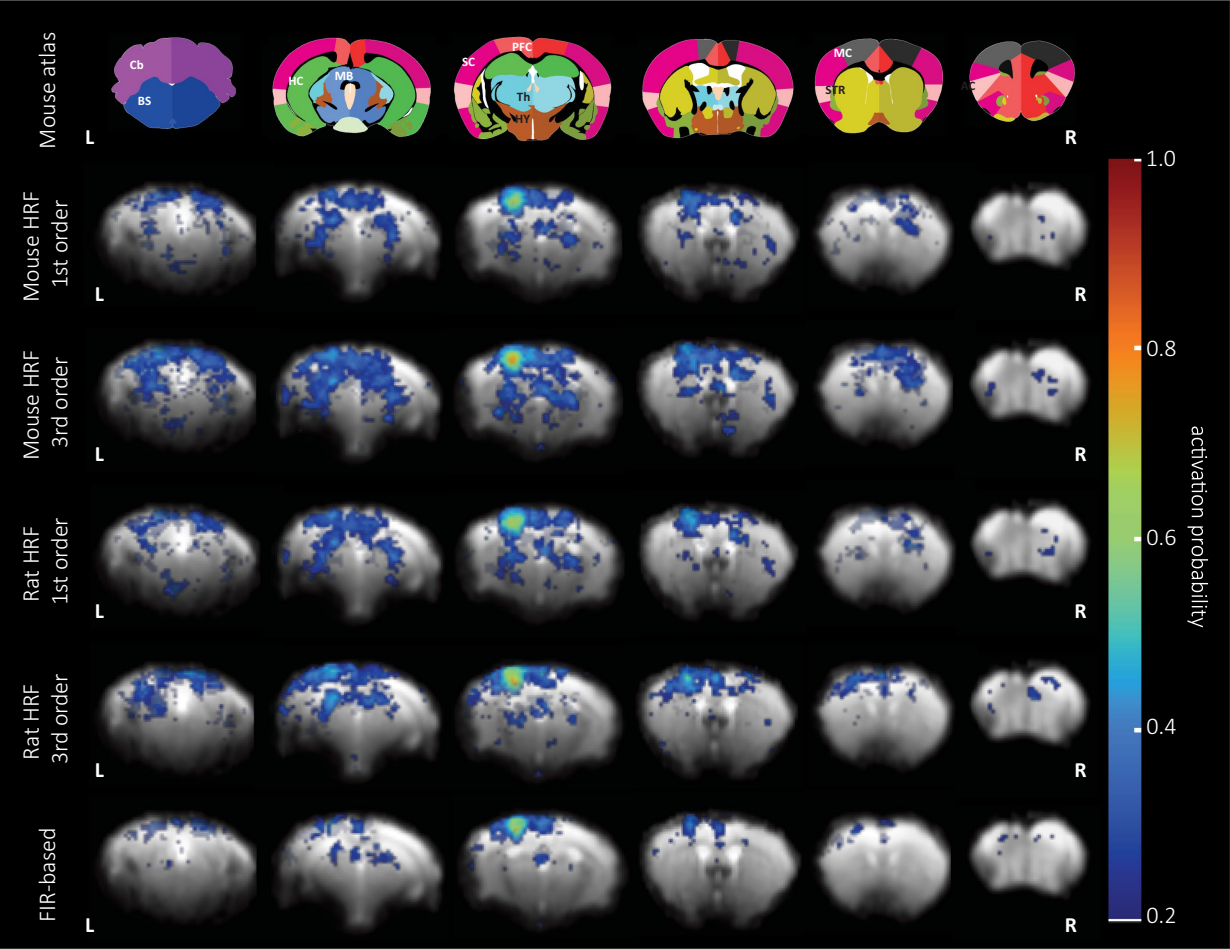
BOLD activation probability in female mice following pinprick stimulation calculated using five different sets of regressors. *Top*: the anatomical atlas consisted of 30 brain structures and was registered to each EPI image. Representative slices are (left to right) −1.72, 0.88, 1.86, 3.34, 4.78, and 5.78 mm from Bregma. GLM analysis combined with the 1^st^ and 3^rd^ order set of the mouse (*rows 2 and 3*) and rat HRF (*rows 4 and 5*), and the 9th order (*bottom row*) of the FIR set was used to calculate the probability map of significantly activated voxels (p_uncorr_ < 0.05) overlaid on an EPI reference image. The area with the highest probability of activation (0.7–0.8) is located in the hind limb cortex of the primary sensory cortex across all the GLM analyses.

To characterize the performance of the mouse HRF implemented in the GLM analysis, we analyzed data from an experimental group with strong BOLD activation (female pp) using GLM combined with the 1^st^ or 3^rd^ order of the canonical set (abbreviated as 1^st^ or 3^rd^ order set/model) using the mouse or rat HRF, or 9^th^ order using the FIR set (Figure 4). Overall, we observed that (1) all GLM analyzes performed equally well in detecting the hind limb cortex of the primary sensory cortex, and that (2) the FIR-based GLM detected fewer activated voxels in the whole brain compared to HRF-based GLM analyzes. We then calculated similarities between the patterns of activated voxels obtained with the 1^st^ order set using the mouse HRF compared to other analyses. The 1^st^ order models using the mouse and rat HRF showed the highest similarity (Dice index: 0.79, Supplementary Figure S5), followed by GLM analyses with that and the 3^rd^ order model using mouse or rat HRF (Dice index: 0.74 / 0.63). The lowest similarity was observed for the FIR-based GLM (Dice index: 0.46).

### Sex differences in mechano-sensory processing

**Figure 5:**
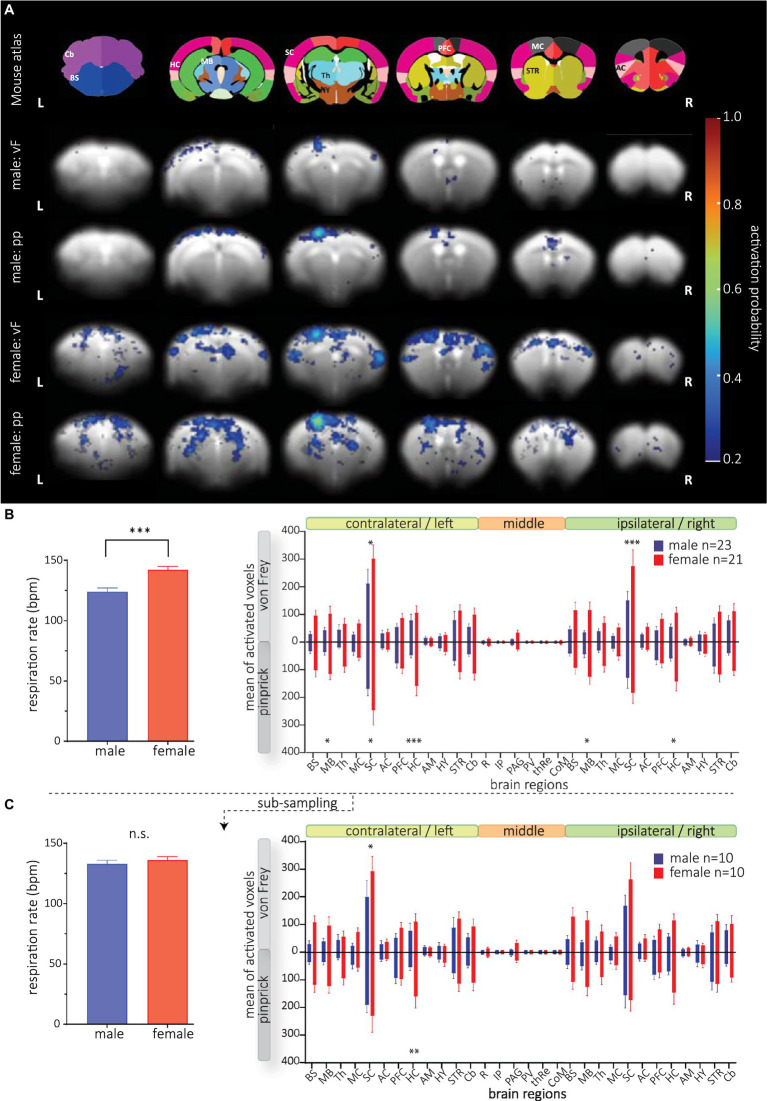
BOLD activation maps following mechanical stimulation in male and female mice. GLM analysis was conducted using the 1^st^ order set with the mouse-specific HRF. (**A**, *top*) The anatomical atlas consisted of 30 brain structures. Activation probability in male (*rows 2 and 3*) and female (*rows 4 and 5*) mice following vF (*rows 2 and 4, respectively*) *and* pp. stimulation (*rows 3 and 5, respectively*). Reliable BOLD activation following mechanical stimulation was detected in contralateral SC, MC and PFC. (**B**, *left*) Respiration rate of male and female animals was significantly different. (*right*) Mean of activated voxels after vF (light gray) and pp. (dark gray) stimulations in male (blue) and female (red), sorted by contralateral and ipsilateral hemispheres and medial brain structures. **(C)** Animals with extreme RR were excluded yielding a subsample of n = 10 animals per group. (Left) RR did not significantly differ in both groups. (right) Significant sex effect for both mechanical stimulus modalities is still observable. Mean of activated voxels after pp. and vF stimulations, sorted by contralateral and ipsilateral hemispheres and medial brain structures after excluding of the outliers. Data are represented as mean ± S.E.M. Asterisks show significant effect between sexes. *p < 0.05, **p < 0.01, ***p < 0.001. BS, brainstem; MB, midbrain; Th, thalamus; MC, motor cortex; SC, sensory cortex; AC, association cortex; PFC, prefrontal cortex; HC, hippocampus; AM, amygdala; STR, striatum; HY, hypothalamus; Cb, cerebellum; R, raphe nucleus; IP, interpeduncular nucleus; PV, paraventricular thalamic nucleus; thRe, nucleus reuniens of the thalamus; PAG, periaqueductal gray; CoM, corpora mammillaria.

We analyzed the effect of vF and pp stimulation on brain-wide activation probability in male and female mice using the 1^st^ order set with the mouse HRF and found reliable BOLD activation following mechanical stimulation in contralateral SC (e.g., S1, S2), MC and PFC (e.g., retrosplenial cortex, cingulate cortex) (Figure 5A). Further, in activation probability maps of female mice, we detected stronger activation in thalamic nuclei and midbrain areas following pp stimulation compared to vF. However, we did not detect a significant effect of the modality used for stimulation [three-way ANOVA, *F*_(1, 2,520)_ = 0.35, *p* = 0.6].

Surprisingly, we found strongly increased numbers of activated voxels in female compared to male mice (Figure 5B, right); this effect was most prominent in the SC. A three-way ANOVA analysis revealed significant differences in sex [*F*_(1, 2,520)_ = 113.0, *p* < 0.0001], and brain structure [*F*_(29, 2,520)_ = 20.34, *p* < 0.0001]. *Post hoc* testing showed that sex differences were most pronounced in the sensory cortex and bilateral midbrain and hippocampus. Analyzing structures that are typically activated by sensory stimulation of the skin, again, we found a strong sex effect [*F*_(1, 1,092)_ = 48.61, *p* < 0.0001] with most pronounced differences in superior colliculus, ventral posteromedial nucleus of the thalamus (VPM), pretectal area (PTA) and zona incerta (Supplementary Figure S6). Furthermore, we found a significant interaction between brain structures and sex [*F*_(29, 2,520)_ = 8.182, *p* < 0.0001] indicating different brain processing of mechanical stimuli (in general) between both sexes. Yet, at this level of analysis, we did not detect interaction between sex and modality [*F*_(1, 2,520)_ = 0.5854, *p* = 0.4].

Since we detected a difference in respiration rate (RR) in both sexes (Figure 5B, right), we wondered whether the RR would affect the GLM performance, thereby causing the observed sex effect. We therefore correlated RR with BOLD amplitudes and numbers of activated voxels in the SC left (Supplementary Figure S7) and found that BOLD amplitudes (male: *r*^2^ = 0.02, *p* = 0.43; female *r*^2^ < 0.0001, *p* = 0.92) and numbers of activated voxels (male: *r*^2^ = 0.13, *p* = 0.05; female *r*^2^ = 0.05, *p* = 0.18) were not affected by RR in mice of both sexes. Since correlation of RR and numbers of voxels in the left SC of male mice indicated a trend, we wondered whether the sex differences were still present if both male and female mice had not significantly different RR. We performed an additional analysis excluding data from animals with slow and fast RR for each group (Figure 5C), yielding group sizes of *n* = 10 with no difference in respiration rate (mean RR of 133 bpm in males and 136 bpm in females, *t*-test, *p* = 0.24 Figure 5C, left). With these two groups, we still found a significant effect of sex following mechanical stimulation [*F*_(1, 1,080)_ = 26.28, *p* < 0.0001] (Figure 5C, right), confirming that RR had no effect on statistical BOLD maps.

Together, these data show different processing of mechanical stimuli between mice of different sex, with female mice showing more activated structures related to sensory input (MB, SC) and learning and memory (HC).

## Discussion

Fundamental to the analysis of task fMRI data in mice is a thorough understanding of stimulus-induced BOLD responses to assure the accuracy, reproducibility and comparability of results. While much work has been done to systematically investigate cortical BOLD responses in rats (Lambers et al., 2020), much less is known in mice. To address this gap, we analyzed BOLD responses in 30 brain structures covering the whole brain in 88 fMRI scans from 30 mice of both sexes following mechanical hind paw stimulation with two different modalities.

### Stimulus-induced HRF is not affected by sex or modality and does not differ between brain structures

Impaired sensation of mechanical stimuli can be a symptom of acute and chronic pain conditions, such as for example neuropathies (Dhandapani et al., 2018). Yet, preclinical fMRI studies using mechanical stimulation modalities are rare in rats (Abaei et al., 2016; Amirmohseni et al., 2016; Just et al., 2020) and—to the best of our knowledge—have not yet been performed in mice. To close this gap, we developed a rotating mechanical stimulator that allows application of calibrated mechanical stimuli with different tools to mice and rats. Following mechanical stimulation with von vF and pp of the plantar hind paw skin, we found similar BOLD responses (and fitted HRFs) across brain structures of the whole brain. This is consistent with previous reports showing no change in the temporal progression of BOLD responses following mechanical or electrical stimulation of the paw skin at noxious and innocuous intensities (Bosshard et al., 2010; Schlegel et al., 2015; Amirmohseni et al., 2016; Lambers et al., 2020). Using different natural sensory stimulus modalities, structural differences in the kinetic of the BOLD response were observed following auditory (Blazquez Freches et al., 2018), visual (Pawela et al., 2008; Bailey et al., 2013) and olfactory stimulation (Chen et al., 2020). However, in most of these studies, the BOLD response strongly depended on the stimulation paradigm, including stimulus duration and frequency. Since we only used a 10 s-stimulation period consistent with our previous experiments in rats (Amirmohseni et al., 2016; Just et al., 2020), we cannot estimate the effect of stimulus duration on the BOLD response. Yet, the impact of the stimulus duration has been previously demonstrated in detail in mice and rats for other modalities and might be similar to mechanical stimulation (Schlegel et al., 2015; Lambers et al., 2020). In our study, we did not exceed a stimulation frequency of 1 Hz to minimize wind-up phenomena in wide-dynamic range neurons of the spinal cord during noxious stimulation (Herrero et al., 2000), which would complicate data interpretation.

Consistent to our results, other studies reported no structural differences in temporal progression of BOLD time courses following whisker (Devonshire et al., 2012) or visual stimulation (Niranjan et al., 2016; Dinh et al., 2020), showing that the stimulation modality (or rather the specifically activated brain structures), *per se*, does not determine the kinetic of the BOLD response. In fact, the BOLD time courses shown in those studies were similar to ours, which may be related to the same anesthetic regimen employed. Different anesthetic agents are known to strongly impact HRF and this effect may as well differ across brain structures (Schlegel et al., 2015). Accordingly, structural differences in BOLD time courses were described for example with medetomidine or propofol alone in mice (Schroeter et al., 2014; Schlegel et al., 2015). However, most anesthetic regimens, including combined MED/ISO (as used in our study), ISO alone, and combined fentanyl/midazolam/isoflurane resulted in a BOLD time course consisting of a single peak (e.g., Schroeter et al., 2014; Sharp et al., 2015; Pradier et al., 2021; You et al., 2021). Yet those using ketamine-based anesthetic regimen showed more complex BOLD time courses with double peaks (Zhao et al., 2020; You et al., 2021).

Finally, we found that the progression of the BOLD response was not affected by sex, which agrees with studies in rats (Lambers et al., 2020; Wang et al., 2020). Based on these studies, there seems to be consistency in mice and rats, at least for the age range early after adolescence (two–four months of age). This is also consistent with humans, where studies with larger cohorts did not detect sex differences in temporal progression of the BOLD response (Squair et al., 2020; Leacy et al., 2022). It is important to mention that our study did not compare BOLD response amplitudes, which differ in men and women (Levin et al., 1998; Cowan et al., 2000; Kaufmann et al., 2001).

Together, these data suggest, that our whole-brain mouse HRF can be used to analyze BOLD responses following sensory stimulation of the skin in mice of both sexes, and that this HRF can very likely to be also applied to different sensory modalities as long as similar anesthetic conditions and stimulation protocols are employed.

### Robustness of GLM performance despite significant differences in implemented HRFs

Understanding the dynamic of the HRF under the respective experimental conditions is a prerequisite for GLM-based BOLD response analysis. Because the HRF is affected by numerous factors, including species, anesthetic regimen, or stimulation protocol, many studies used custom-made HRF for GLM analysis (Schroeter et al., 2014; Jung et al., 2019; Lambers et al., 2020; Pradier et al., 2021; You et al., 2021). However, this comes at the cost of reproducibility and comparability across different studies. Therefore, we compared the GLM performance of two HRFs: one obtained from the rat sensory cortex (Lambers et al., 2020), and one derived from the mouse whole-brain. While the mouse HRFs showed significantly slower kinetics (slower time to peak, larger half-width and delayed undershoot) compared to the rat HRF, the results of the GLM performed with both HRFs on the same data set were similar. Yet, they were not identical, and it is plausible that these differences between both modeled signals could significantly affect GLM analysis if the BOLD signals had higher amplitudes or were acquired with faster image acquisition schemes as it is used for single-slice GE-EPI (Gil et al., 2021) or line-scanning BOLD fMRI (Yu et al., 2014; Albers et al., 2018; Choi et al., 2022). Accordingly, we recommend employing a species-specific HRF when using HRF-based GLM analysis for BOLD detection by implementation of the HRF parameters in the software package that is being used to make future studies more comparable.

### Divergent BOLD activation following mechanical stimulation in male and female mice

We detected remarkable sex-differences in brain activation following vF and pp stimulation. These brain structures were part of SC, HC and MB in both hemispheres. This activation pattern is reminiscent to what has been described in a previous study investigating acute inflammatory pain in male and female rats. Using manganese-enhanced MRI, the authors found increased activation of S1, M1, CPu and amygdala in female compared to male rats (Malheiros et al., 2021). Moreover, investigating muscle and cutaneous pain in humans, Henderson and colleagues found increased activation of mid-cingulate cortex and dorsolateral PFC in naïve female compared to male human volunteers, while the overall pain intensity rating was not affected (Henderson et al., 2008). Other examples for divergent BOLD activation following noxious stimuli in human volunteers have also been observed following noxious visceral and thermal stimulation using fMRI (Linnman et al., 2012; Wang et al., 2019). While growing evidence shows that female individuals have greater sensitivity to pain in humans and animals compared to their male counterparts (Mogil, 2020), the behavioral or phenotypic manifestation of these differences may be subtle and dependent on the number of subjects used. In fact, differences in heat thresholds have been reported in naïve male and female mice with group sizes of approximately *n* = 4,000 (Mogil and Chanda, 2005). While this difference in somatosensation/nociception was highly significant, the effect size was so small that a sample size calculation predicted a significant difference between both groups with at least 450 animals per group. Since we believe it is reasonable to expect equally small effect sizes in mechanosensation/mechanical pain processing, this suggests that most studies are under-powered to detect sex-differences, and hence, a concerted effort across different labs will be needed to establish these differences at the behavioral level with sufficient statistical power. While we did not determine mechanical thresholds/sensitivity in awake animals in the present study to avoid stress induced by behavioral testing (i.e., sitting on a mesh floor and repeated von Frey and pinprick stimulation), parallel behavioral and fMRI studies could investigate sex differences in more detail in the future.

Furthermore, as shown in this study and by others before, the sex differences in brain processing appear magnitudes larger than the behavioral/cognitive phenotype, thereby unveiling potentially divergent mechanisms in cerebral pain processing in naïve humans and animals of both sexes. A likely mechanism for different cerebral processing could involve the descending pain inhibition, which exerts different effects in male and female volunteers (Popescu et al., 2010), and which has also been replicated in animal fMRI studies (Silva et al., 2018). However, these results are not entirely consistent, especially not in more recent studies in human volunteers (Vollert et al., 2022) and need further investigation.

### Potential limitations of BOLD fMRI to detect sex differences

Despite the statistical significance of the observed sex differences in cerebral activation following mechanical stimulation, several confounding factors could contribute to this effect. First, the different respiration rates (RR) in male and female mice could hint at physiological differences between both sexes that could contribute to the observed BOLD differences. In such a scenario, a changed RR could potentially alter blood pCO_2_ levels that would affect vasodilation and also the amplitude of measured BOLD response (Van Alst et al., 2019). Since we did not detect significant correlations between both factors, our data suggest that different breathing patterns are not a major contributor to the observed sex differences. This is also in line with previous reports that did not detect differences in blood gas composition in male and female rats (Bavis et al., 2006). However, future studies should investigate the stability of physiological parameters in male and female mice over time to more comprehensively assess the contribution of these factors on BOLD fMRI.

Secondly, the analgesic/sedative effects of MED could differently engage neuronal circuits in male and female mice. Although the analgesic and sedative properties of MED have been differentiated from each other and have been assigned to be mediated by different brain structures (Buerkle and Yaksh, 1998), both effects are more challenging to dissect when the drug is given systemically. Following a subcutaneous route of administration—at a concentration very similar to the one used in our study—the analgesic effects of MED coincide with the impairment of motor performance (Pertovaara et al., 1990; Idanpaan-Heikkila et al., 1994), making the estimation of its analgesic effect virtually impossible. However, other studies suggest that fewer α2-adrenergic receptors are needed to mediate the analgesic compared to the sedative effect of MED (Hayashi et al., 1995), suggesting in turn a state of MED-mediated analgesia under our experimental conditions. However, unfortunately only few studies have investigated sex differences of α2- adrenergic receptor agonists on analgesia. While clonidine has been shown to have greater analgesic properties in males compared to female rats (Kiefel and Bodnar, 1992), tizanidine did not exert different analgesic properties in naïve male and female rats (Rodríguez-Palma et al., 2022). It is important to mention that both drugs and MED differ in their pharmacokinetic profile (Buerkle and Yaksh, 1998; Kawamata et al., 2003), which makes a direct comparison difficult. In conclusion, we cannot rule out a sex-specific effect of MED, which will be investigated in future studies.

## Conclusion

We found no differences in HRFs of mice across all different experimental conditions and computed a whole-brain mouse HRF, which is based on 88 functional scans from 30 animals. This mouse-specific HRF showed significantly slower kinetics than a previously reported rat-derived HRF. Based on these findings, we recommend using a species-specific HRF for analysis of task BOLD fMRI data. Finally, we detected strong differences in cerebral BOLD activation in male and female mice after innocuous and noxious mechanical stimulus thereby exposing divergent processing of mechanical stimuli between both sexes.

## Data availability statement

The raw data supporting the conclusions of this article will be made available by the authors, without undue reservation.

## Ethics statement

The animal study was reviewed and approved by the State Agency for Nature, Environment and Consumer Protection North Rhine-Westphalia (LANUV).

## Author contributions

H-FC and BP performed the experiments, wrote the manuscript, and created figures and tables. DS, BP, and EP-Z wrote the ethical application. H-FC, HL, NN, and BP performed data analysis. MS designed the mechanical stimulator. BP, CF, and EP-Z conceived, designed, and supervised the study. All authors reviewed the article.

## Funding

This work was supported by the Interdisziplinäres Zentrum für klinische Forschung grant (IZKF, Pog2/027/20) to EP-Z, a Deutsche Forschungsgemeinschaft grant (DFG, Fa474/6) to CF, an Innovative medizinische Forschung grant (IMF, I-PR121913) to BP, and the support of the imaging core unit PIX of the IZKF.

## Conflict of interest

The authors declare that the research was conducted in the absence of any commercial or financial relationships that could be construed as a potential conflict of interest.

## Publisher’s note

All claims expressed in this article are solely those of the authors and do not necessarily represent those of their affiliated organizations, or those of the publisher, the editors and the reviewers. Any product that may be evaluated in this article, or claim that may be made by its manufacturer, is not guaranteed or endorsed by the publisher.

## Footnotes

^1^https://www.nitrc.org/projects/mricrogl/

^2^https://github.com/TheFaberLab/functional_t-test
