# Supplementary material for: Generation of a whole-brain hemodynamic response function and sex-specific differences in cerebral processing of mechano-sensation in mice detected by BOLD fMRI
Source: Front Neurosci. 2023 Aug 28;17:1187328. doi: 10.3389/fnins.2023.1187328 (PMC10493293; doi:10.3389/fnins.2023.1187328)
Supplement: Supplementary file 1 [file Data_Sheet_1.PDF]

Supplement table 1: a composition of 30 brain regions including both hemisphere in the digital mouse atlas

Abbrevation	Area	Hemisphere
BS le	Brain Stem	left
BS ri	Brain Stem	right
R	raphe nucleus	m
MB le	Midbrain	left
MB ri	Midbrain	right
IP	interpeduncular nucleus	m
PAG	periaqueductal gray	m
Th le	Thalamus	left
Th ri	Thalamus	right
PV	paraventricular thalamic nucleus	m
thRem	reuniens thalamic nucleus	m
MC le	Motor cortex	left
MC ri	Motor cortex	right
SC le	Sensory cortex	left
SC ri	Sensory cortex	right
AC le	association Cortex	left
AC ri	association Cortex	right
PFC le	Prefrontal cortex	left
PFC ri	Prefrontal cortex	right
HC le	Hippocampus	left
HC ri	Hippocampus	right
AM le	Amygdala	left
AM ri	Amygdala	right
HY le	Hypothalamus	left
HY ri	Hypothalamus	right
CoM	corpora mammillaria	m
STR le	Striatum	left
STR ri	Striatum	right
Cb le	cerebellum	left
Cb ri	cerebellum	right

Suppl. Tab. 2: p-values of functional t-test between regions across 4 groups. Threshold for minimum group size is n<6. p-values from brain structures with n ≤ 5 are not shown in the table. Adjusted threshold for significance corrected for multiple testing is p<0.0002.

number of HRF	Region	BS le	BS ri	MB le	MB ri	PAG	Th le	Th ri	MC le	MC ri	SC le	SC ri	AC le	AC ri	PFC le	PFC ri	HC le	HC ri	STR le	STR ri	Cb le	Cb ri
14	BS le																					
18	BS ri	0.2195																				
21	MB le	0.0468	0.6384																			
21	MB ri	0.0892	0.6306	0.5203																		
10	PAG	0.0217	0.0607	0.6821	0.1254																	
11	Th le	0.2318	0.1379	0.5060	0.0662	0.2421																
12	Th ri	0.1678	0.6863	0.7079	0.8152	0.4259	0.3986															
25	MC le	0.2999	0.4569	0.5458	0.4558	0.3108	0.4264	0.8325														
9	MC ri	0.7379	0.4305	0.2335	0.3897	0.2775	0.3123	0.7840	0.7834													
54	SC le	0.4030	0.3567	0.2210	0.1182	0.2027	0.3311	0.1441	0.4782	0.5955												
33	SC ri	0.0637	0.8273	0.4953	0.2104	0.0774	0.2979	0.4777	0.2167	0.2429	0.2361											
16	AC le	0.8527	0.7344	0.4148	0.2239	0.1895	0.5799	0.6029	0.9679	0.8710	0.8557	0.5119										
12	AC ri	0.3504	0.3519	0.8437	0.4213	0.7044	0.7609	0.5492	0.9556	0.7193	0.6845	0.3887	0.9015									
42	PFC le	0.1864	0.5806	0.5882	0.2919	0.2469	0.7405	0.3393	0.4166	0.3806	0.6930	0.8155	0.5334	0.7074								
16	PFC ri	0.4929	0.9203	0.8282	0.4871	0.1882	0.4445	0.7950	0.7425	0.6996	0.5957	0.8920	0.9364	0.6607	0.8900							
32	HC le	0.7644	0.6954	0.2013	0.3538	0.1565	0.2454	0.2812	0.8723	0.8354	0.9059	0.2149	0.9844	0.6691	0.5789	0.9512						
28	HC ri	0.0807	0.7653	0.7518	0.3744	0.1126	0.3257	0.5852	0.5626	0.2698	0.0691	0.6911	0.6026	0.4979	0.3550	0.9130	0.3284					
15	STR le	0.3245	0.1965	0.0856	0.1532	0.2018	0.1084	0.6537	0.4849	0.9838	0.0960	0.0785	0.7107	0.1834	0.1174	0.4852	0.3855	0.0872				
20	STR ri	0.2999	0.9660	0.9152	0.8279	0.2440	0.4271	0.6575	0.7168	0.5265	0.7124	0.7270	0.6910	0.7420	0.7690	0.8969	0.8045	0.8684	0.2796			
27	Cb le	0.4360	0.8213	0.5047	0.3629	0.1261	0.2049	0.8500	0.7152	0.7881	0.4086	0.5206	0.9444	0.4924	0.4045	0.9955	0.8986	0.5313	0.3890	0.7242		
30	Cb ri	0.8728	0.4227	0.2114	0.1176	0.1601	0.5800	0.3466	0.8986	0.8938	0.7077	0.2330	0.9969	0.7949	0.5704	0.7751	0.8102	0.3201	0.4841	0.5821	0.5969	
	p-values	BS le	BS ri	MB le	MB ri	PAG	Th le	Th ri	MC le	MC ri	SC le	SC ri	AC le	AC ri	PFC le	PFC ri	HC le	HC ri	STR le	STR ri	Cb le	Cb ri

Suppl. Tab. 3: p-values of functional t-test between two regions in male animals during pinprick (bottom triangle) and von Frey (top triangle) stimulation. Threshold for minimum group size is n<6. p-values from brain structures with n ≤ 5 are not shown in the table. Adjusted threshold for significance corrected for multiple testing is p<0.005 (pinprick) and p<0.008 (von Frey).

number of HRF	Pinprick	BS le	BS ri	MB le	MB ri	PAG	Th le	Th ri	MC le	MC ri	SC le	SC ri	AC le	AC ri	PFC le	PFC ri	HC le	HC ri	STR le	STR ri	Cb le	Cb ri	von Frey	number of HRF
n=2	BS le																						BS le	n=3
n=3	BS ri																						BS ri	n=3
x	MB le																						MB le	n=4
n=4	MB ri																						MB ri	n=4
x	PAG																						PAG	x
n=2	Th le																						Th le	n=1
n=3	Th ri																						Th ri	n=1
n=4	MC le																						MC le	n=3
x	MC ri																						MC ri	n=1
n=13	SC le											0.7743					0.8399					0.2417	SC le	n=13
n=7	SC ri										0.3718						0.9691					0.4933	SC ri	n=6
n=1	AC le																						AC le	n=3
n=1	AC ri																						AC ri	n=3
n=10	PFC le										0.5583	0.8748											PFC le	n=4
n=5	PFC ri																						PFC ri	n=1
n=6	HC le										0.4879	0.1112			0.0773	0.1425						0.4165	HC le	n=7
n=6	HC ri										0.6414	0.7554			0.5284	0.7106	0.2432						HC ri	n=5
n=2	STR le																						STR le	n=4
n=5	STR ri																						STR ri	n=1
n=5	Cb le																						Cb le	n=4
n=5	Cb ri																						Cb ri	n=7
	**p value**	BS le	BS ri	MB le	MB ri	PAG	Th le	Th ri	MC le	MC ri	SC le	SC ri	AC le	AC ri	PFC le	PFC ri	HC le	HC ri	STR le	STR ri	Cb le	Cb ri	**p value**	

Suppl. Tab. 4: p-values of functional t-test between two regions in female animals during pinprick (bottom triangle) and von Frey (top triangle) stimulation. Threshold for minimum group size is n<6. Brain structures with n ≤ 5 are not shown in the table. Adjusted threshold for significance corrected for multiple testing is p<0.0006 for both groups.

number of HRF	Pinprick	BS le	BS ri	MB le	MB ri	PAG	Th le	Th ri	MC le	MC ri	SC le	SC ri	AC le	AC ri	PFC le	PFC ri	HC le	HC ri	STR le	STR ri	Cb le	Cb ri	von Frey	number of HRF
n=4	BS le																						BS le	n=5
n=6	BS ri			0.6117	0.7373				0.7843		0.7394	0.7409	0.7315		0.8101		0.7813	0.4917		0.7167	0.5719	0.0816	BS ri	n=6
n=8	MB le		0.8515		0.8433				0.5940		0.2775	0.5686	0.6280		0.4259		0.4371	0.7098		0.9324	0.2046	0.2322	MB le	n=9
n=6	MB ri		0.8649	0.7763					0.4776		0.0865	0.3011	0.3589		0.5728		0.1782	0.3173		0.6025	0.0530	0.1316	MB ri	n=7
n=5	PAG																						PAG	n=5
n=4	Th le																						Th le	n=4
n=4	Th ri																						Th ri	n=4
n=7	MC le		0.8107	0.8639	0.5061						0.6919	0.6281	0.9719		0.9841		0.8548	0.4846		0.7349	0.5182	0.5577	MC le	n=11
n=4	MC ri																						MC ri	n=4
n=14	SC le		0.8014	0.6521	0.5012				0.8396			0.3427	0.9105		0.8497		0.8750	0.0992		0.4490	0.8129	0.2457	SC le	n=14
n=9	SC ri		0.6802	0.7675	0.5823				0.6049		0.9875		0.6267		0.3868		0.4967	0.8208		0.7832	0.2702	0.0621	SC ri	n=11
n=5	AC le														0.9958		0.9702	0.5359		0.5996	0.5801	0.6290	AC le	n=7
n=5	AC ri																						AC ri	n=3
n=13	PFC le		0.5970	0.8005	0.7301				0.4673		0.8695	0.9745					0.9804	0.1943		0.5024	0.6687	0.2652	PFC le	n=15
n=6	PFC ri		0.8032	0.7836	0.8249				0.6296		0.5343	0.5857			0.7946								PFC ri	n=4
n=11	HC le		0.7776	0.9010	0.7694				0.7344		0.9978	0.9876			0.9593	0.8245		0.5110		0.6644	0.8219	0.2780	HC le	n=8
n=9	HC ri		0.9964	0.6752	0.6041				0.8534		0.8729	0.7767			0.6875	0.7155	0.8822			0.7893	0.2726	0.1830	HC ri	n=8
n=4	STR le																						STR le	n=5
n=6	STR ri		0.8151	0.9684	0.9049				0.8888		0.7701	0.8871			0.9113	0.9005	0.9814	0.7785			0.4447	0.4128	STR ri	n=8
n=12	Cb le		0.9700	0.7137	0.4233				0.7397		0.6491	0.8167			0.7951	0.5000	0.8492	0.9650		0.7411		0.6155	Cb le	n=6
n=10	Cb ri		0.7018	0.3645	0.1311				0.5298		0.7637	0.8774			0.8153	0.1915	0.6437	0.7230		0.4637	0.4812		Cb ri	n=8
	**p value**	BS le	BS ri	MB le	MB ri	PAG	Th le	Th ri	MC le	MC ri	SC le	SC ri	AC le	AC ri	PFC le	PFC ri	HC le	HC ri	STR le	STR ri	Cb le	Cb ri	**p value**	
