# Supplementary material for: Generation of a whole-brain hemodynamic response function and sex-specific differences in cerebral processing of mechano-sensation in mice detected by BOLD fMRI
Source: Front Neurosci. 2023 Aug 28;17:1187328. doi: 10.3389/fnins.2023.1187328 (PMC10493293; doi:10.3389/fnins.2023.1187328)
Supplement: Supplementary file 2 [file Data_Sheet_2.PDF]

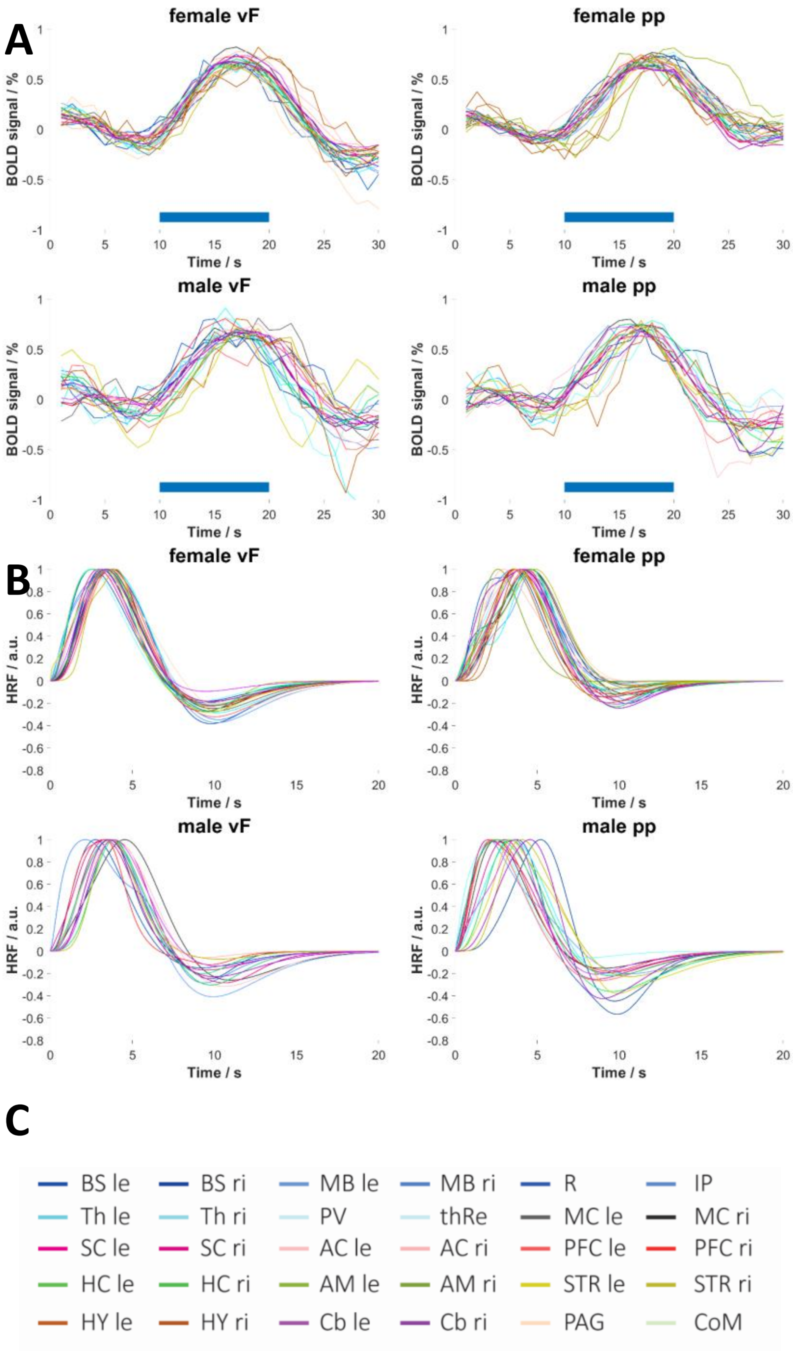

Supplement figure 1. (A) Averaged BOLD responses per group. After extraction of time courses and determination of positively activated voxels, individual time courses of each structure were measured per animal and then averaged for females (*middle*) and males (*bottom*) following vF (*left*) and pp (*right*) stimulation. (B) Normalized mean HRF per group. The time course of BOLD responses was fitted to the convolution of the canonical HRF and the stimulation paradigm. From these fits, HRFs were calculated for each brain structure per animal and averaged for each group. Blue horizontal bar demonstrates the onset and duration of the stimulation. (C) Each colored line shows an individual brain structure according to the mouse atlas. Bilateral structures: BS: brainstem, MB: midbrain, Th: thalamus, MC: motor cortex, SC: sensory cortex, AC: association cortex, PFC: prefrontal cortex, HC: hippocampus, AM: amygdala, STR: striatum, HY: hypothalamus, Cb: cerebellum, le: left, ri: right, medial structures: R: raphe nucleus, IP: interpeduncular nucleus, PV, paraventricular thalamic nucleus, thRe: nucleus reuniens of the thalamus, PAG: periaqueductal gray, CoM: corpora mammillaria.

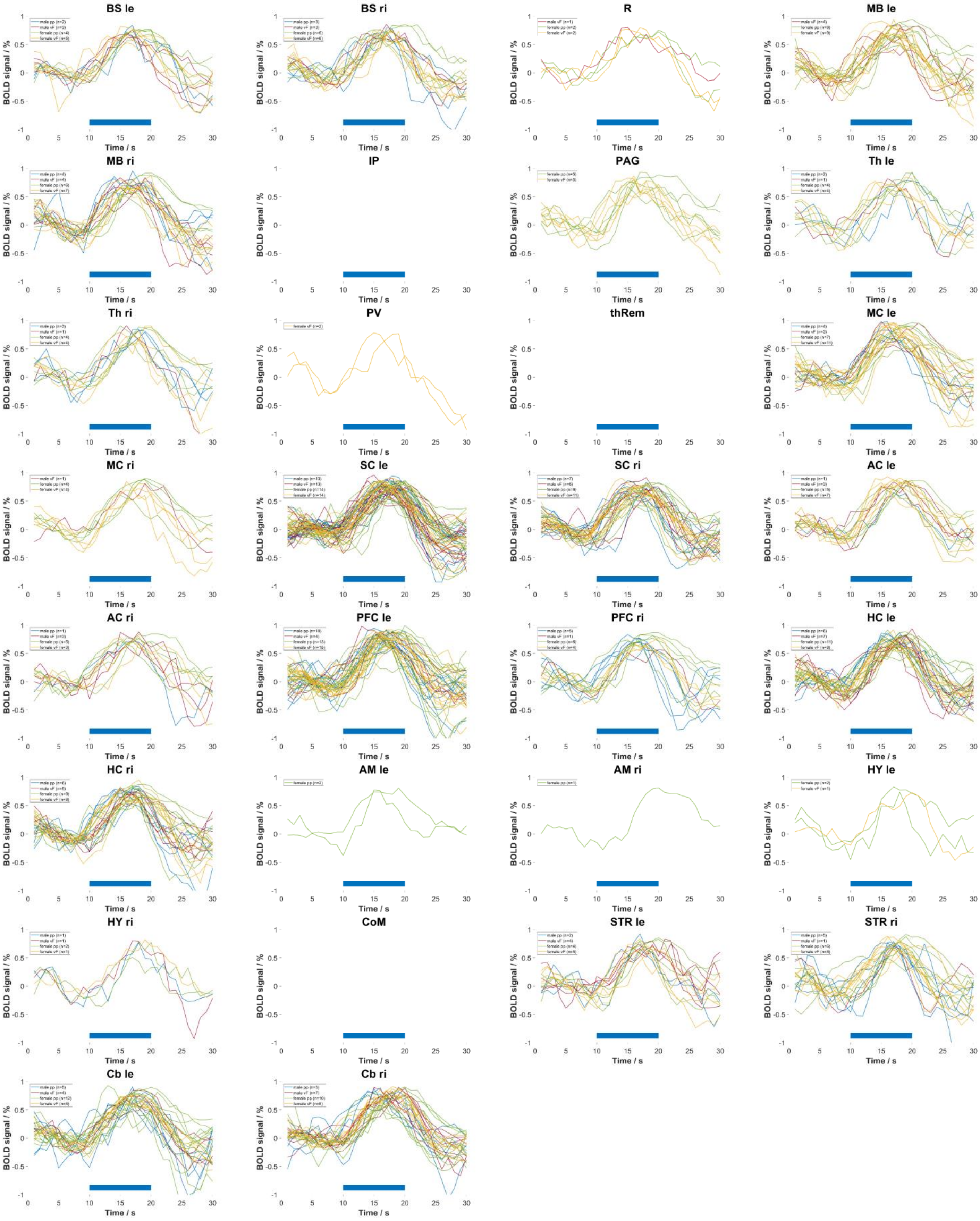

Supplement figure 2. BOLD time course per region. After extraction of time courses and determination of positively activated voxels, individual time courses of each structure were measured per animal. Blue horizontal bar demonstrates the onset and duration of the stimulation. Colored lines show individual HRFs due to groups: male_pp: blue, male_vF: red, female_pp: green, female_vF: yellow.

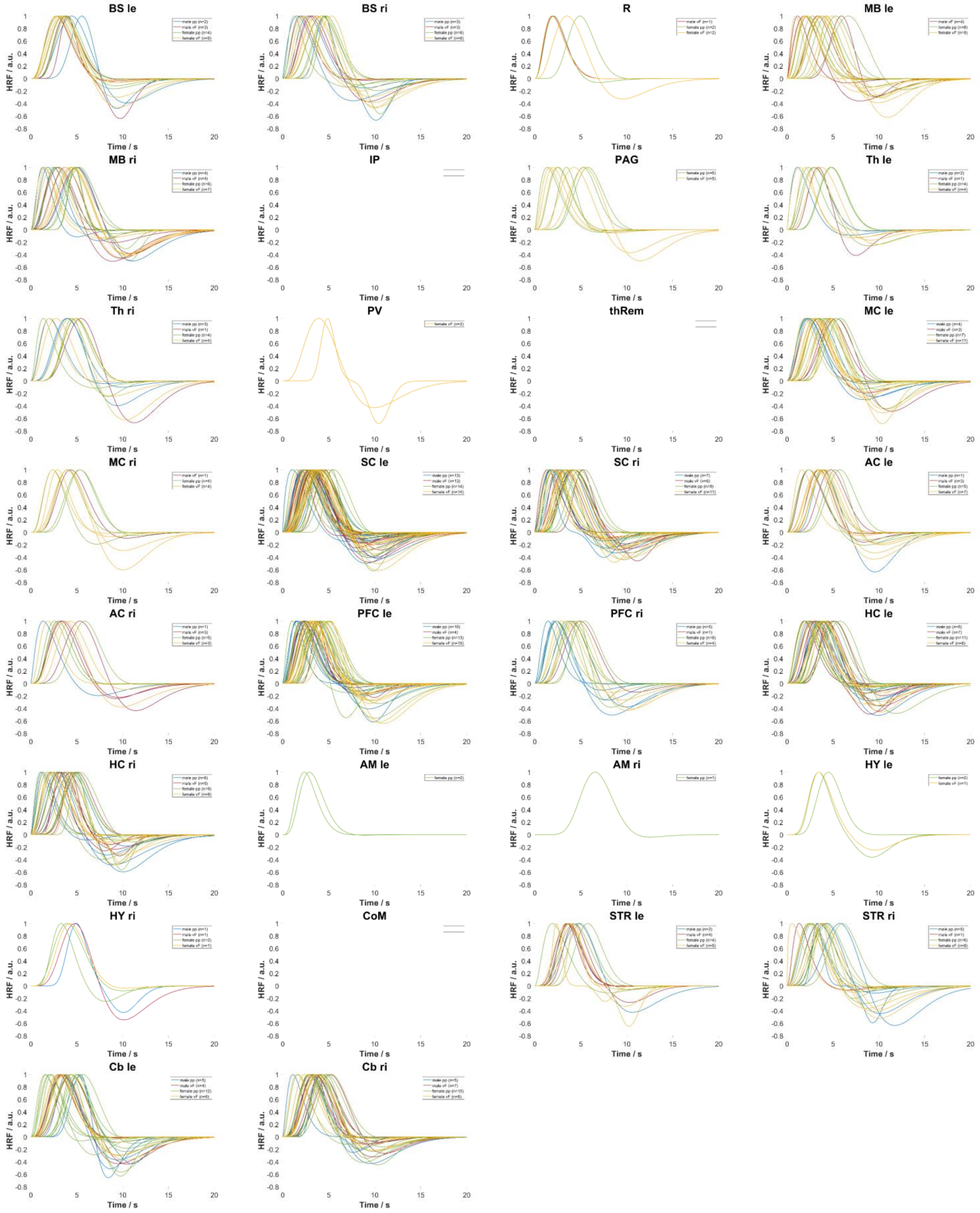

Supplement figure 3. Individual HRFs per region. The time course of BOLD responses was fitted to the convolution of the canonical HRF and the stimulation paradigm. From these fits, HRFs were calculated for each brain structure per animal. Colored lines show individual HRFs due to groups: male_pp: blue, male_vF: red, female_pp: green, female_vF: yellow.

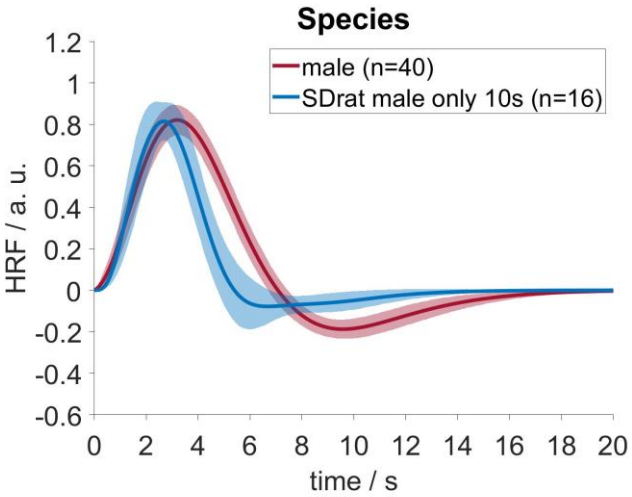

Supplement figure 4: comparison of HRFs from sensory cortex between mice and rats following mechanical stimulation. Solid lines indicate mean values, shaded areas represent confidence intervals.

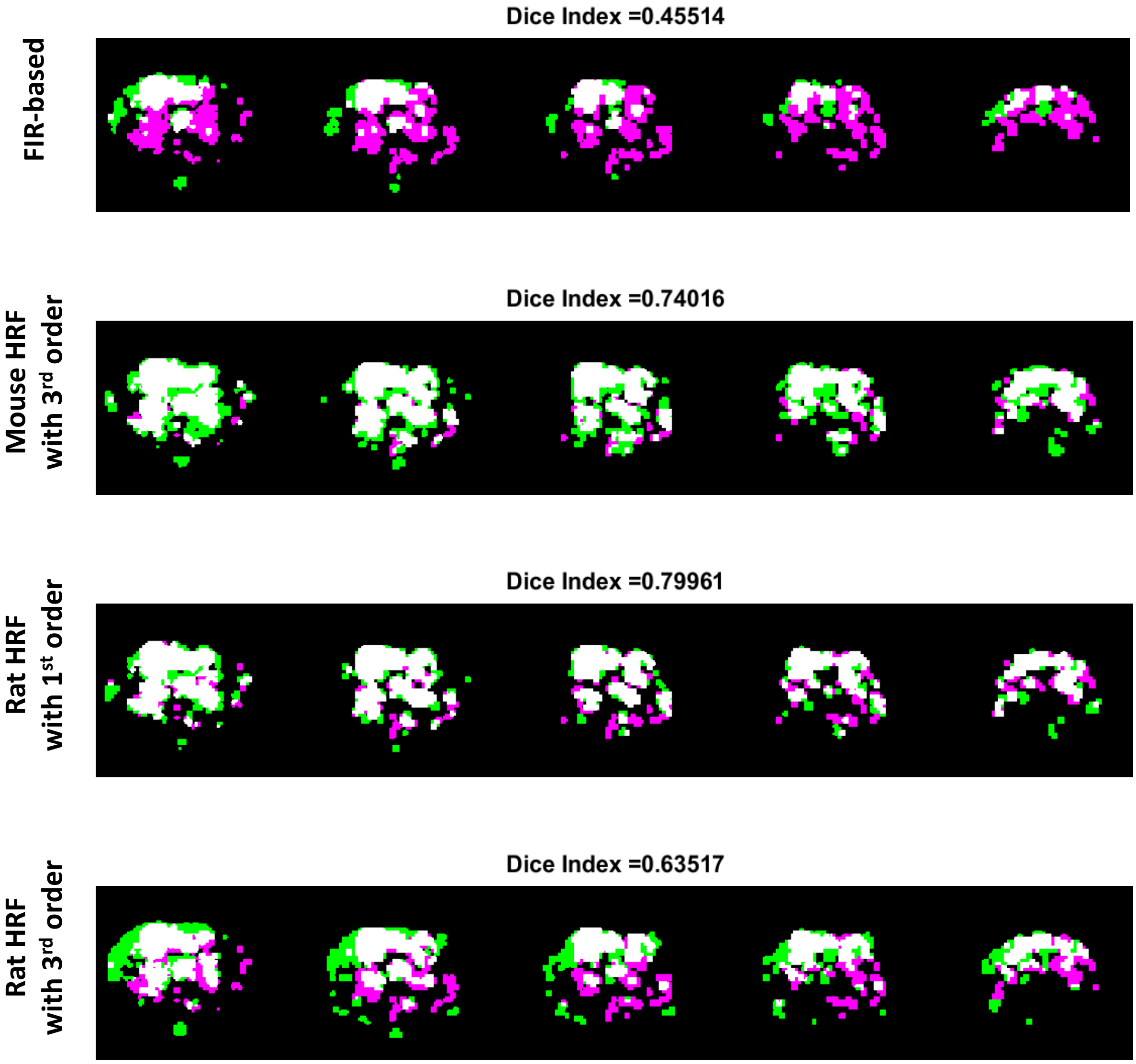

Supplement figure 5: Similarity between GLM with the 1st order of mouse HRF compared to different GLM models as investigated by dice index.

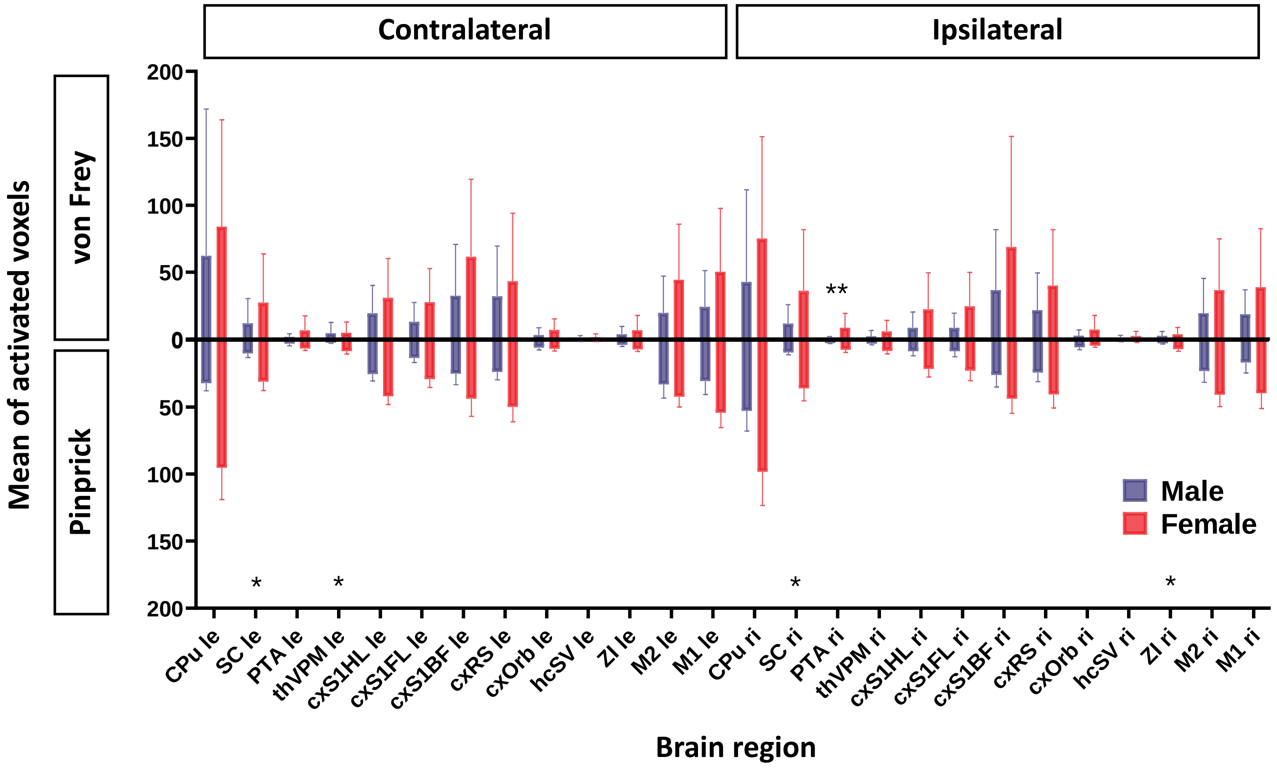

Supplement figure 6: Mean of activated voxels in specific brain structures after pp and vF stimulations, sorted by brain structures of contralateral and ipsilateral hemispheres. Bilateral structures: CPu: caudate putamen, SC: superior colliculus, PTA: pretectal area, thVPM: ventral posteromedial thalamic nucleus, cxS1HL: primary somatosensory cortex hindlimb, cxS1FL: primary somatosensory cortex forelimb, cxS1BF: primary somatosensory cortex barrel field, cxRS: retrosplenial cortex, cxOrb: orbital cortex, hcSV: ventral subiculum, ZI: zona incerta, M1: primary motor cortex, M2: secondary motor cortex, le: left, ri: right. Data are represented as mean ± S.E.M.

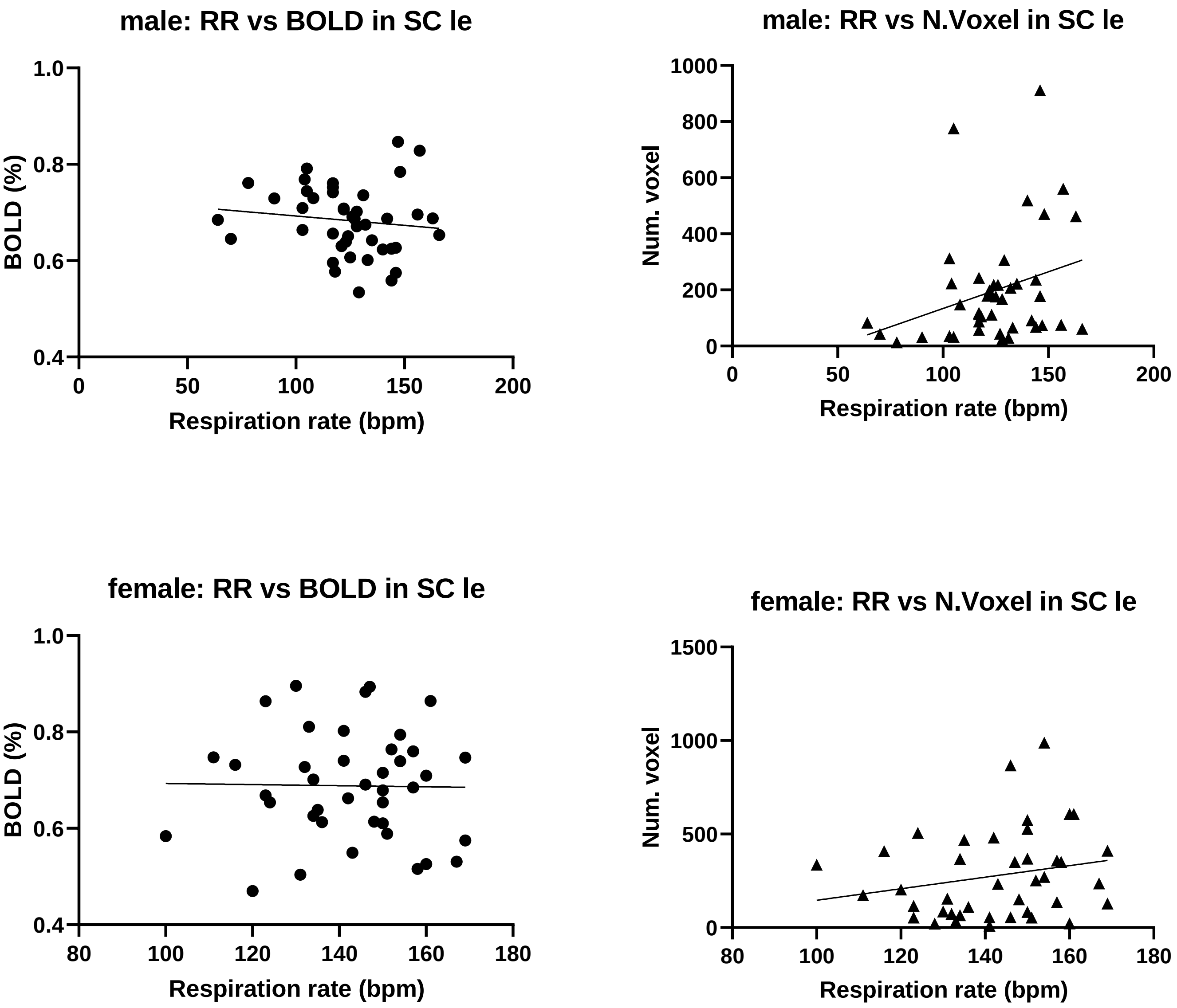

Supplement figure 7: No significant correlation between respiration rate and parameters of the BOLD response. Correlation of respiration rate (RR) with BOLD amplitudes (left) and numbers of activated voxels in the contralateral sensory cortex (right).
